# Rational, yet simple, design and synthesis of an antifreeze-protein inspired polymer for cellular cryopreservation†
†Electronic supplementary information (ESI) available. See DOI: 10.1039/c5cc04647e



**DOI:** 10.1039/c5cc04647e

**Published:** 2015-07-15

**Authors:** Daniel E. Mitchell, Neil R. Cameron, Matthew I. Gibson

**Affiliations:** a Department of Chemistry , University of Warwick , Coventry , CV4 7AL , UK . Email: m.i.gibson@warwick.ac.uk; b MOAC Doctoral Training Centre , University of Warwick , Coventry , CV4 7AL , UK; c Department of Materials Science and Engineering , Monash University , Australia; d School of Engineering , University of Warwick , UK

## Abstract

Antifreeze (glyco) proteins AF(G)Ps are potent ice recrystallization inhibitors, which is a desirable property to enhance cryopreservation of donor tissue/cells. Here we present the rational synthesis of a new, biomimetic, ice-recrystallization inhibiting polymer derived from a cheap commodity polymer, based on an ampholyte structure. The polymer is used to enhance the cryopreservation of red blood cells, demonstrating a macromolecular solution to tissue storage.

## Abstract 2

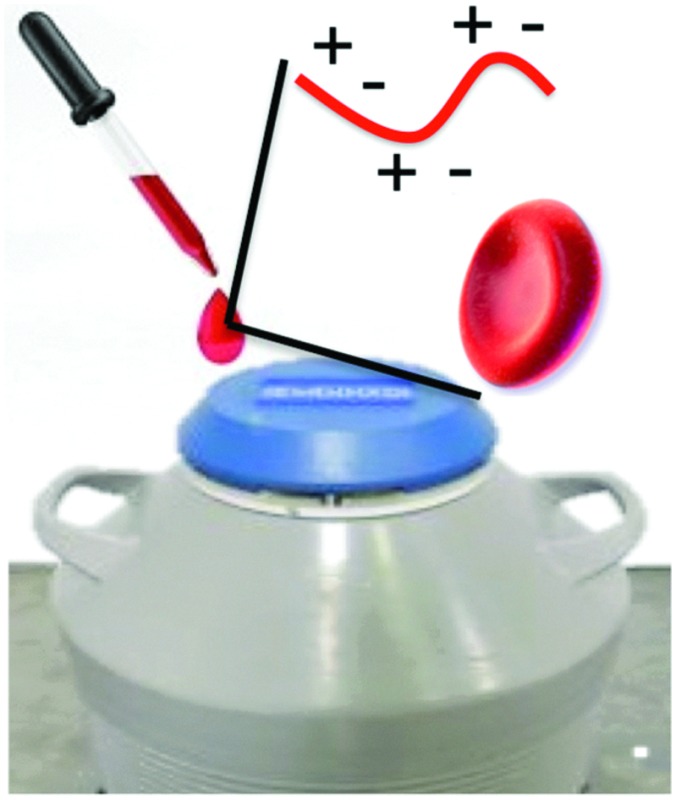
A structurally simple synthetic polymer based on alternating charged side chains is designed and synthesised to mimic antifreeze proteins. The polymer is found to enhance the cryopreservation of red blood cells.

In the freezing polar regions of the Earth, nature has evolved a number of methods to survive. One of these is freeze avoidance^1^ (as opposed to freeze tolerance)^2^ using antifreeze proteins (AFPs) or antifreeze glycoproteins (AF(G)Ps). Ever since they were discovered in polar fish species AF(G)Ps have attracted interest due to their ability to interact with ice^3^ and their potential applications in cryopreservation, cryosurgery, food-storage, anti-icing surfaces and more.^4–7^ AF(G)Ps have three key properties; (i) thermal hysteresis (TH) – the non-colligative depression of the freezing point, which does not affect the equilibrium melting point;^8^ (ii) dynamic ice shaping (DIS) where the shape of ice crystals is altered due to binding to specific faces on the ice surface;^9^ (iii) ice recrystallization inhibition (IRI) whereby the rate of ice crystal growth (Ostwald ripening) is slowed.^10^ IRI is a particularly interesting property as it has been found that ice recrystallization during thawing is a major contributor to cell death.^11,12^

New cryoprotectants are urgently needed to improve the availability of cells/tissues/organs to address the needs of a growing and ageing population. For example, blood can only be stored for a maximum of 42 days and stock quantities vary over the course of a year, meaning that there is always a shortage.^13^ Emerging regenerative medicine therapies based on stem cells also require efficient cryostorage. The current state-of-the-art cryoprotection involves the addition of large amounts of organic solvents such as glycerol or DMSO. Whilst successful, these solvents can cause cellular toxicity and, ideally, should not be directly transfused. There are also some cell types for which no effective cryopreservation solution exists.

Considering the above, there have been several attempts at cryopreservation using AF(G)Ps but these have met with mixed results. For example, addition of AFP to erythrocytes gave some cryopreservation enhancement, but above a critical concentration, it actually decreased viability.^14^ This was found to be due to the formation of needle-like (spicular) ice crystals due to the DIS/TH activity of AF(G)Ps. There are several other studies demonstrating both benefits and problems of AF(G)Ps in cryopreservation, with the problems normally due to ice shaping, which have prevented their application.^15,16^ Furthermore there is some evidence that AF(G)Ps can be toxic to human cells.^17^ In 2003 Ben and co-workers demonstrated that short glycopeptides could specifically reproduce IRI, but not TH/DIS suggesting that there may be a synthetic route to new cryoprotectant molecules.^18^ Several glycopeptides, glycopolymers and even small molecules have since been identified with varying degrees of IRI activity.^19–22^ Some of these are still relatively challenging to synthesize and their cryopreservation activity is still under investigation. As an alternative both Gibson and coworkers and Inada *et al.* have separately investigated synthetic polymers as AF(G)P mimics, in particular the use of poly(vinyl alcohol) (PVA) which has strong IRI activity,^23,24^ with very few other polymers reported with this unique property. Polymers are easy to access on a large scale, are highly tunable in terms of composition and architecture and are widely used in personal care and pharmaceutical industries making them appealing additives. Addition of just 0.1 wt% of PVA was found to enhance red blood cell cryopreservation by inhibiting ice growth.^25^ Matsumura and coworkers identified that carboxyl-modified poly(ε-lysine) could enhance stem cell cryopreservation although the mechanism was unclear.^26^ Gibson and co-workers used controlled radical polymerization to generate well defined poly(ampholytes) with both amino and carboxyl side chains with definite IRI activity but that a 1 : 1 ratio of cationic to anionic groups was required for maximum activity and unambiguously demonstrated that IRI was (in part) the cryoprotective mechanism of poly(ampholytes).^27^ To enable exploitation of IRI active polymers, there still exists a need to identify new materials, ensure their availability in quantities needed for application and to demonstrate their biological potential.

This manuscript describes an easy and accessible synthetic route to new poly(ampholytes) with definite IRI activity, derived from the bulk commodity polymer poly(methyl vinyl ether-*alt*-maleic anhydride) (Gantraz™) widely used in the coatings industry. The synthetic route ensures perfectly alternating cationic/anionic charges to maximize activity (as opposed to previous routes). This new material was then shown to dramatically enhance red blood cell cryopreservation enabling solvent-free storage of this crucial component of modern medicine.

Previously polyampholytes have been prepared by post-polymerisation functionalization of poly(amines) which intrinsically gives rise to a statistical distribution of the charged groups.^28^ Considering Nature requires explicit control over sequence distribution in AFGPs, but tolerates molecular weight distribution, we devised a synthetic strategy to match this. The low cost (<$20 kg^–1^), but structurally defined, commodity polymer PMVEMA (*M*_w_ = 311 kDa) contains alternating anhydrides in the main side chain, providing an opportunity to obtain alternating polymers *via* ring-opening, but has large dispersity as it is obtained by free-radical polymerization (as with most commodity-polymers). The poly(ampholyte) was prepared by nucleophilic ring-opening of the anhydride ring with *N*-Boc-ethanolamine, followed by TFA deprotection in high yield with a minimum of synthetic steps, Fig. 1. Following exhaustive dialysis, conversion of the anhydrides to esters was confirmed by IR spectroscopy and quantitative incorporation of the ethanolamine group was determined by ^1^H NMR (Fig. 2).

**Fig. 1:**
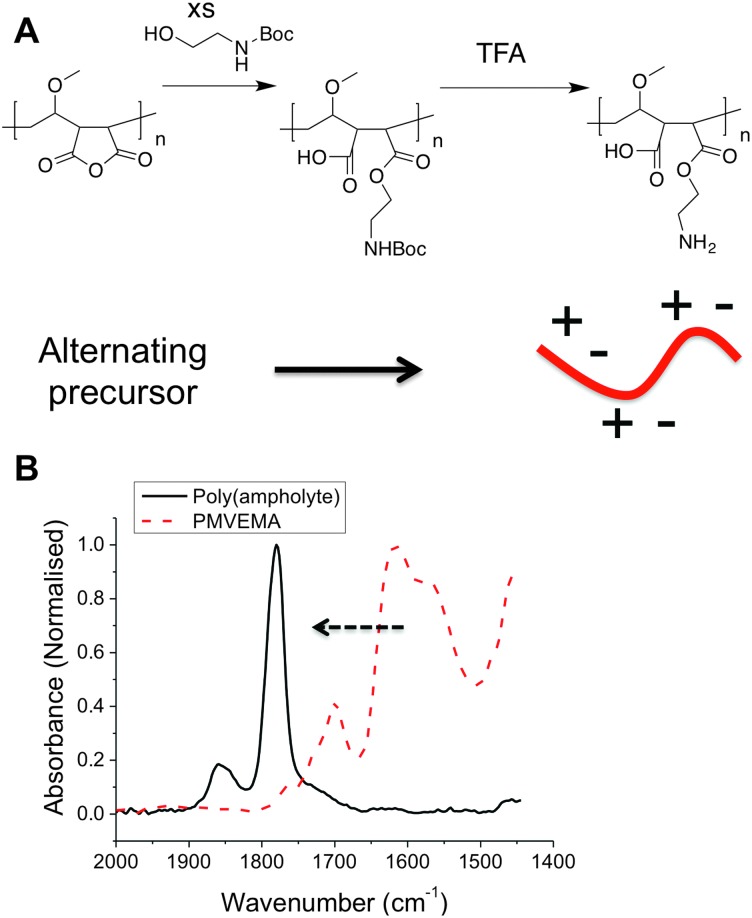
Synthesis of new poly(ampholyte) by ring opening of the maleic acid units. (A) Synthetic route employed; (B) IR spectra showing conversion of anhydride into carboxylic acid and ester, confirming substitution.

**Fig. 2:**
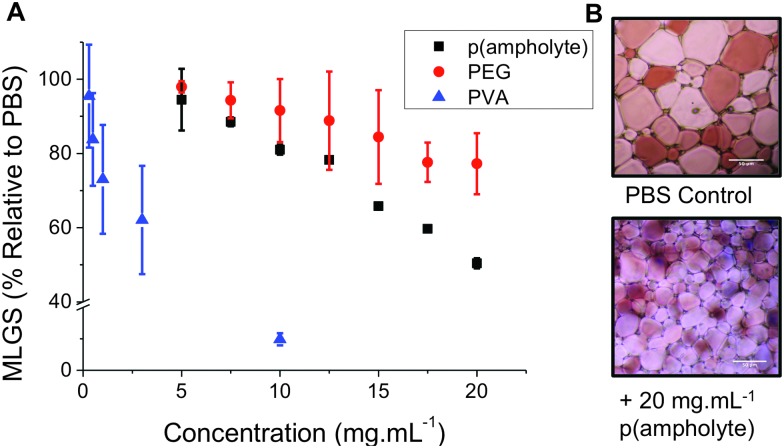
Ice recrystallization inhibition of PMVEMA-*co*-EA. (A) Comparison with PEG (100 kDa) as a negative control and PVA_10_ as a positive. Mean largest grain size is expressed as a percentage of PBS buffer. Error bars represent ± SD from a minimum of 3 repeats; (B) example micrographs of ice wafers with and without polymeric IRI agent. Both images are of equal magnification, scale bars represent 50 μm.

With this new, low cost, poly(ampholyte) to hand it was necessary to evaluate its IRI activity. A modified splat test was employed, which involves creating a wafer of small ice crystals, which are then allowed to grow at –8 °C, and then their average crystal sizes are measured, relative to a control (PBS). Small MLGS (mean largest grain size) values indicate increased IRI activity. As a negative control we included a high molecular weight (100 kDa) poly(ethylene glycol), PEG. This PEG was chosen to ensure that any viscosity-related effects (due to high molecular weight) do not bias our measurements. In keeping with reports of other poly(ampholytes), this new polymer was capable of inhibiting 50% of ice growth at 20 mg mL^–1^. Whilst this is less active than our positive control, PVA, it is still a definite effect and somewhat surprising given the huge structural differences between this polymer and Native AF(G)Ps, and represents one of only a handful of synthetic polymers with this activity. It also highlights the current lack of understanding or the key structural motifs required for IRI and the need to understand the underlying mechanisms. Inhibiting ice growth at a concentration of 20 mg mL^–1^ is also relatively low in a cryopreservation context: up to 40 wt% (∼400 mg mL^–1^) is required for tradition organic solvents applied in cryopreservation. The *N*-Boc precursor had no observable IRI and had limited aqueous solubility.

Our previous studies have shown that addition of PVA to red blood cells dramatically reduces ice-induced damage, but that the level of cell recovery using PVA alone was relatively low (<40%).^25^ To improve this, hydroxyethyl starch (HES) was selected as a co-cryopreservative – it is non-penetrating macromolecules and does not lead to vitrification, unlike *e.g.* DMSO. HES is used clinically as a plasma expander, so is ideally suited to blood-contacting applications. Cytocompatibilty of the poly(ampholyte) was evaluated by incubation with fresh ovine red blood cells (RBCs) for 4 hours, after which the degree of haemolysis was measured, Fig. 3A (cell recovery = 1/haemolysis, see ESI†). At concentrations up to 40 mg mL^–1^ (above what is needed in later experiments) there was no statistically significant indication of haemolysis, indicating the polymers were compatible with RBCs. At concentrations as high as 100 mg mL^–1^, haemolysis was observed, as would be expected for almost any foreign additive at such high concentrations. At these very high (>50 mg mL^–1^) concentrations the polymer solution also becomes very viscous, which might contribute to apparent toxicity, due to the challenge of separating undamaged cells and hence there is the opportunity for false negative results. To assess the polymers' cryopreservation enhancement, RBCs were prepared in an isotonic cryopreservation solution containing HES, and different amounts of the poly(ampholyte) were added. Cells were frozen by rapid cooling to –196 °C (liquid nitrogen) and storage in nitrogen vapour for 5 days. Following this time the cells were thawed at room temperature for 1 hour. These thawing conditions are crucial – ice recrystallization is maximised under slow thawing conditions (the property being probed here) and is representative of large volume cryopreservation (*e.g.* blood bags, or organs) where thermal gradients across the sample lead to ice growth. Control experiments using fast thawing were also conducted, which improves recovery in these low volumes but is challenging in a ‘real world’ situation (ESI†). It should be noted that differential scanning calorimetry confirmed no vitrification occurred during freezing. The results of the cryopreservation are shown in Fig. 3B.

**Fig. 3:**
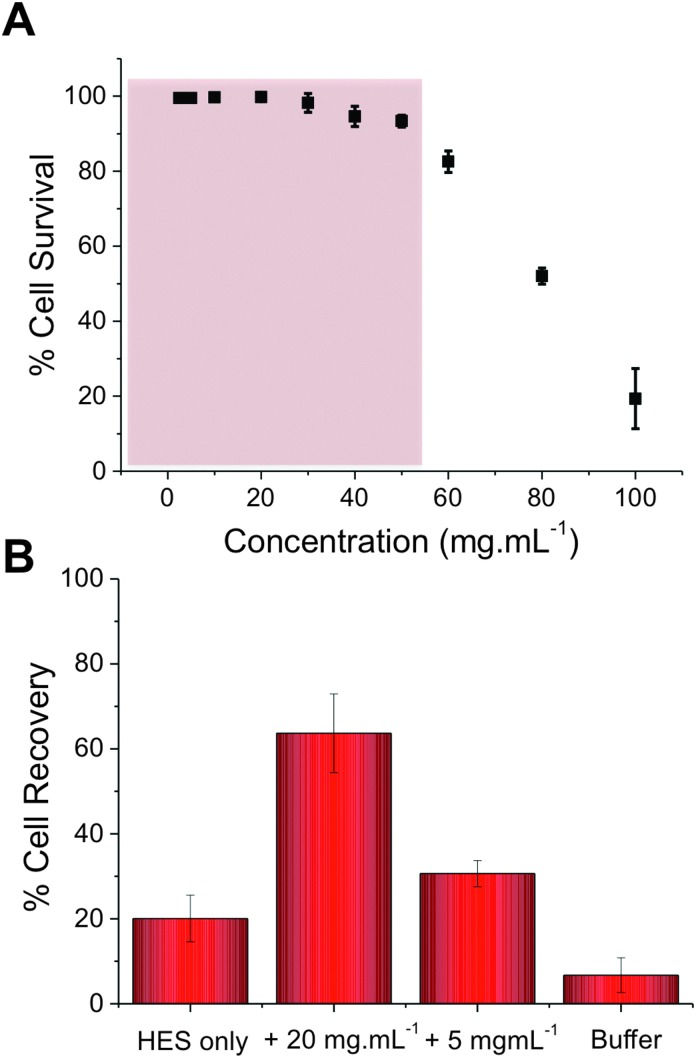
Recovery of red blood cells when slow-thawed on the bench top. (A) Cytocompatibility assay of RBCs with p(ampholyte) and the working range highlighted in red. Incubation time = 4 hours; (B) cell recovery after freezing (–196 °C, 5 days) and thawing (room temperature, 1 hour). Recovery is expressed as a fraction of an unfrozen positive control, error bars represent ± SD from a minimum of 3 repeats.

Using HES alone, only 20% of the RBCs were recovered. However, addition of 5 mg mL^–1^ poly(ampholyte) increased this to 30%, and addition of 20 mg mL^–1^ increased it to an impressive 60%. Higher concentrations could not be tested due to the increased solution viscosity in the cryopreservation solution. The dose-dependence of the cryopreservation correlates well with the observed IRI activity of the polymers and the dose required is far less than was required for a lysine-based poly(amphotyle) when cryopreserving stem cells,^26^ which needed >100 mg mL^–1^ for cryopreservation. It should again be highlighted that by using slow-thawing conditions, we are maximising potential for cell damage to provide a rigours test – higher levels of recovery could be achieved with fast thawing (∼80%) but our aim is to eliminate all recrystallization associated damage. Considering the ease, and scale, of the polymer synthesis and its clear beneficial effect on improving blood cryopreservation this demonstrates that biomimics of AF(G)Ps can have a huge impact on improving the availability of donor cells. The work presented here also demonstrates that the rational design of ice-modifying macromolecules, tuned to their specific application is possible. In the long term, this will enable us to fully understanding how macromolecules (synthetic and biological) control crystallisation processes. Future work will extend to a wide range of cell types and also obtain structure–function relationships to enable an understanding of why certain polymers can inhibit ice growth.

We have demonstrated a new, facile, biomimetic approach to engineering ice-recrystallization inhibiting polymers, inspired by antifreeze proteins. A region-regular poly(ampholyte) was synthesized from a bulk commodity precursor and shown to have specific ice recrystallization inhibition activity. The poly(ampholyte) was non-toxic to red blood cells and facilitated a remarkable enhancement in the post-freezing cell recovery by inhibiting ice growth during thawing. This work shows that the identification, and translation of new cryopreservatives is possible, using synthetic, as opposed to biological approaches. It is also a step towards the ability to routinely mimic these proteins, which have a complex function, which will bring benefits both to cryopreservation (and other fields) but to our understanding of interfacial process at ice crystals.

Equipment used was supported by the Innovative Uses for Advanced Materials in the Modern World (AM2), with support from Advantage West Midlands (AWM) and part funded by the European Regional Development Fund (ERDF). This work was supported by a Research Project Grant from the Royal Society. DEM acknowledges the EPSRC for a studentship from the MOAC Doctoral Training Centre; grant number EP/F500378/1.

## References

[cit1] Cheng C. C. and DeVries A. L., in Life Under Extreme Conditions, ed. G. di Prisco, Springer Berlin Heidelberg, 1991, ch. 1, pp. 1–14 10.1007/978-3-642-76056-31.

[cit2] Storey K. B., Storey J. M. (1988). Physiol. Rev..

[cit3] DeVries A. L. (1988). Comp. Biochem. Physiol., Part B: Biochem. Mol. Biol..

[cit4] Harding M. M., Anderberg P. I., Haymet A. D. J. (2003). Eur. J. Biochem..

[cit5] Esser-Kahn A. P., Trang V., Francis M. B. (2010). J. Am. Chem. Soc..

[cit6] Muldrew K., Rewcastle J., Donnelly B. J., Saliken J. C., Liang S., Goldie S., Olson M., Baissalov R., Sandison G. (2001). Cryobiology.

[cit7] Griffith M., Ewart K. V. (1995). Biotechnol. Adv..

[cit8] Celik Y., Graham L. A., Mok Y. F., Bar M., Davies P. L., Braslavsky I. (2010). Proc. Natl. Acad. Sci. U. S. A..

[cit9] Houston Jr M. E., Chao H., Hodges R. S., Sykes B. D., Kay C. M., Sönnichsen F. D., Loewen M. C., Davies P. L. (1998). J. Biol. Chem..

[cit10] Czechura P., Tam R. Y., Dimitrijevic E., Murphy A. V., Ben R. N. (2008). J. Am. Chem. Soc..

[cit11] Deller R. C., Vatish M., Mitchell D. A., Gibson M. I. (2014). Nat. Commun..

[cit12] Fowler A., Toner M. (2006). Ann. N. Y. Acad. Sci..

[cit13] Ali A., Auvinen M. K., Rautonen J. (2010). Transfusion.

[cit14] Carpenter J. F., Hansen T. N. (1992). Proc. Natl. Acad. Sci. U. S. A..

[cit15] O'Neil L., Paynter S. J., Fuller B. J., Shaw R. W., DeVries A. L. (1998). Cryobiology.

[cit16] Wang T., Zhu Q., Yang X., Layne Jr J. R., Devries A. L. (1994). Cryobiology.

[cit17] Liu S., Wang W., von Moos E., Jackman J., Mealing G., Monette R., Ben R. N. (2007). Biomacromolecules.

[cit18] Eniade A., Murphy A. V., Landreau G., Ben R. N. (2001). Bioconjugate Chem..

[cit19] Balcerzak A. K., Ferreira S. S., Trant J. F., Ben R. N. (2012). Bioorg. Med. Chem. Lett..

[cit20] Corcilius L., Santhakumar G., Stone R. S., Capicciotti C. J., Joseph S., Matthews J. M., Ben R. N., Payne R. J. (2013). Bioorg. Med. Chem..

[cit21] Capicciotti C. J., Leclère M., Perras F. A., Bryce D. L., Paulin H., Harden J., Liu Y., Ben R. N. (2012). Chem. Sci..

[cit22] Gibson M. I., Barker C. A., Spain S. G., Albertin L., Cameron N. R. (2009). Biomacromolecules.

[cit23] Congdon T., Notman R., Gibson M. I. (2013). Biomacromolecules.

[cit24] Inada T., Modak P. R. (2006). Chem. Eng. Sci..

[cit25] Deller R. C., Vatish M., Mitchell D. A., Gibson M. I. (2014). Nat. Commun..

[cit26] Matsumura K., Hyon S.-H. (2009). Biomaterials.

[cit27] Mitchell D. E., Lilliman M., Spain S. G., Gibson M. I. (2014). Biomater. Sci..

[cit28] Gauthier M. A., Gibson M. I., Klok H.-A. (2009). Angew. Chem., Int. Ed..

