# Supplementary material for: Rational, yet simple, design and synthesis of an antifreeze-protein inspired polymer for cellular cryopreservation
Source: Chem Commun (Camb). 2015 Jul 15;51(65):12977–80. doi: 10.1039/c5cc04647e (PMC4672748; doi:10.1039/c5cc04647e)
Supplement: Supplementary file 1 [file CC-051-C5CC04647E-s001.pdf]

Electronic Supplementary Material (ESI) for ChemComm.
This journal is © The Royal Society of Chemistry 2015

Supplementary Information for

Rational, yet simple, design and synthesis of an antifreeze-protein inspired polymer for cellular cryopreservation

Daniel E. Mitchell[a], [b] Neil R. Cameron[c],[d] and Matthew I. Gibson*[a]

[a] Department of Chemistry, University of Warwick, Gibbet Hill Road, Coventry, CV4 7AL, UK.

[b] MOAC DTC, University of Warwick, Gibbet Hill Road, Coventry, CV4 7AL, UK.

[c] Department of Materials Science and Engineering, Monash University, Australia

[d] School of Engineering, University of Warwick, UK

*Corresponding author: E-mail: m.i.gibson@warwick.ac.uk.

Materials.

Poly(methyl vinyl ether-*alt*-maleic anhydride) (PMVEMA Mw 311 kDa), ethanol amine, Di-*tert*-butyl dicarbonate (BOC), poly(ethylene glycol) (PEG, 100 kDa size), mannitol, hydroxyethyl starch (HES), sodium chloride, tetrahydrofuran and trifluoroacetic acid were purchased from Sigma Aldrich (UK). Ovine red blood cells in Alserver's solution were purchased from TCS biosciences (UK). Phosphate-buffered saline (PBS) solution was prepared using preformulated tablets (Sigma-Aldrich) in 200 mL of Milli-Q water (>18.2 Ω mean resistivity) to give [NaCl] = 0.138 M, [KCl] = 0.0027 M, and pH 7.4.

Physical and analytical methods

^{1}H and ^{13}C NMR spectra were recorded on Bruker DPX-300 and DPX-400 spectrometers using deuterated solvents obtained from Sigma-Aldrich. Chemical shifts in ppm (δ) are reported relative to residual tetramethylsilane (TMS). FTIR spectra were acquired using a Bruker Vector 22 FTIR spectrometer with a Golden Gate diamond attenuated total reflection cell. A total of 128 scans were collected on samples in their native (dry) state. Bright and fluorescence microscopy were performed on an Olympus CKX41 with MoticamPro 205C and CoolLed pE-300-W light source, images analysed using Motic Images Advanced 3.2. Cells used were screened, pathogen-free, defibrinated sheep red blood cells in Alsever's solution. All incubations of red blood cells were visualised at 1/10 dilution in PBS, with 2 μL solution on a glass slide with a glass cover slip. Absorbance was measured on a microplate reader (Synergy HT multi-mode microplate reader, BioTek UK).

Synthesis of *N*-boc ethanolamine.

3.57g Di-*tert*-butyl dicarbonate was added to 1g ethanolamine (1:1 molar ratio) and reacted at room temperature for 2 hours, producing *N*-boc ethanolamine. ^{1}H NMR ($CDCl_3$): δ 4.92 (br, 1H –ROH); δ 3.74 (br, 2H –OCH_2); δ 3.51 (s, 1H -CH_2NHCOO); δ 3.32 (br 2H– CH_2CH_2NH); δ 1.67 (br, 9H $OC(CH_3)_3$). Mass Spectrometry: m/z (EI^-) calc for $(CH_3)_3COCONHCH_2CH_2OH$, 161.2; found 160.2 $[M]^-$.

Functionalization of PMVEMA with *N*-Boc ethanolamine. 2 g of *N*-boc-ethanolamine was added to 0.4 g PMVEMA (Mw 311 kDa) dissolved in 10 mL THF. The mixture was warmed to 55°C and allowed to react for 24 hours. This was then dialysed against deionized water (7 water changes 48 hours) and lyophilized. Functionalization was confirmed by NMR and FTIR spectroscopy. ^{1}H NMR (D_2O): δ 3.66 (br 2H –OCH_2CH_2); δ 3.47 (br 3H -OCH_3); δ 3.32 (br H –CH_2NHCOO); δ 2.91-3.12 (br 2H CHCH(COO)CH)); δ 2.62 (br 2H CH_2CH_2NH); δ 1.92 (br 2H -$CHCH_2CH$); δ 1.28 (br 9H -$OC(CH_3)_3$).

Removal of BOC protecting group. 0.3 g N-Boc functional-PMVEMA dissolved in methanol was treated with 2 mL trifluoroacetic acid. The mixture was reacted at room temperature for 3 hours. This was followed by dialysis (7 water changes, 48 hours) against deionized water and lyophilisation. ^{1}H spectroscopy was used to determine functionalization. ^{1}H NMR (D_2O): δ 3.66 (br 2H –OCH_2CH_2); δ 3.47 (br 3H -OCH_3); δ 3.32 (br 2H –CH_2NHCOO); δ 2.91- 3.12 (br 2H CHCH(COO)CH)); δ 2.62 (br 2H CH_2CH_2NH); δ 1.92 (br 2H -$CHCH_2CH$);

Ice recrystallisation inhibition (splat) assay.

Ice recrystallisation inhibition was measured using a modified splay assay.[1] A 10 μL sample of polymer dissolved in PBS buffer (pH 7.4) was dropped 1.40 m onto a chilled glass coverslip sat on a piece of polished aluminium placed on dry ice. Upon hitting the chilled glass coverslip, a wafer with diameter of approximately 10 mm and thickness 10 μm was formed instantaneously. The glass coverslip was transferred onto the Linkam cryostage and held at −6 °C under N_2 for 30 minutes. Photographs were obtained using an Olympus CX 41 microscope with a UIS-2 20×/0.45/∞/0–2/FN22 lens and crossed polarizers (Olympus Ltd, Southend on sea, UK), equipped with a Canon DSLR 500D digital camera. Images were taken of the initial wafer (to ensure that a polycrystalline sample had been obtained) and after 30 minutes. Image processing was conducted using Image J, which is freely available. In brief, ten of the largest ice crystals in the field of view were measured and the single largest length in any axis recorded. This was repeated for at least three wafers and the average (mean) value was calculated to find the largest grain dimension along any axis. The average of this value from three individual wafers was calculated to give the mean largest grain size (MLGS). This average value was then compared to that of a PBS buffer negative control providing a way of quantifying the amount of IRI activity.

Heamolysis Testing

Samples containing 250 µL red blood cells (RBCs) and 250 µL of polymer solution were incubated at 37 °C for 30 minutes prior to testing. After centrifugation to concentrate down the RBCs, 10 µL of the supernatant was added to 90 µL of PBS buffer in a 96 well plate. The absorbance was measured at 450 nm and compared against a PBS buffer positive control and a deionised water negative control to determine the cell survival rate. RBCs leach heme into the supernatant when they die, providing an easily available method of testing cell survival rate. Samples were tested as 6 repeats.

Cryopreservation of RBCs.

A 250 µL aliquot of prepared RBCs (packed cell volume between 32 and 52 %) was added to 250 µL of a standard cryosolution (containing HES (350 $mgml^{-1}$), mannitol (30 $mgml^{-1}$) sodium chloride (6.5 $mgml^{-1}$)) and vortexed gently to ensure mixing. pMVEMA-*co*-EA was added at various concentrations to this cryosolution in order to determine whether any beneficial effect could be seen. These were then rapidly frozen in triplicate by immersion into liquid nitrogen and stored within a liquid nitrogen container for 5 days. Thawing was either performed at 42 °C for 2 minutes in a water bath, or on the bench top for 1 hour.

Measurement of Red Blood Cell haemolysis and cell recovery.

A 250 µL aliquot of red blood cell (RCB) / cryoprotectant solution was centrifuged in a 1.5 ml Eppendorf tube for 5 minutes at 6000 rpm. Then 10 µL of the supernatant

was removed and added to 90 µL of PBS in a well of a 96-well plate. Absorbance was measured at 450 nm and compared to an unfrozen positive control containing 250 µL prepared RCBs and 250 µL PBS buffer. 100 % hemolysis samples were prepared by adding 250 µL RCBs to distilled water. Cell recovery was calculated by subtracting the attained haemolysis (%) from 100 (%) giving a figure for cell recovery (%).

Additional Data

^{1}H NMR spectrum of *N*-boc ethanolamine

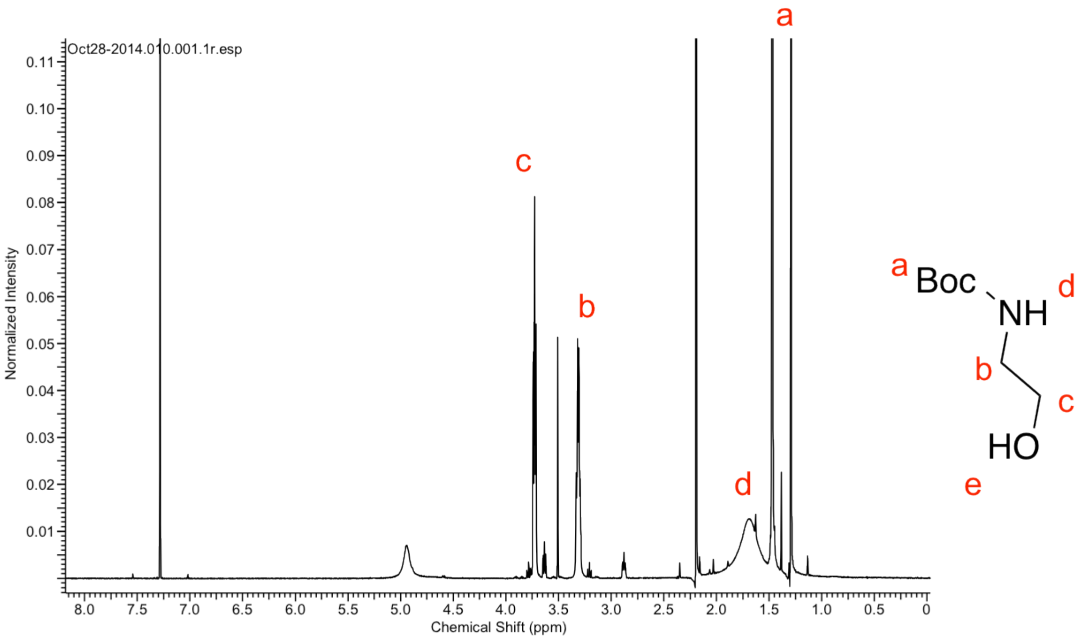

Figure S1. ^{1}H NMR of N-boc ethanolamine (400 MHz, $CDCl_3$).

Mass-spectroscopy spectrum for *N*-boc ethanolamine

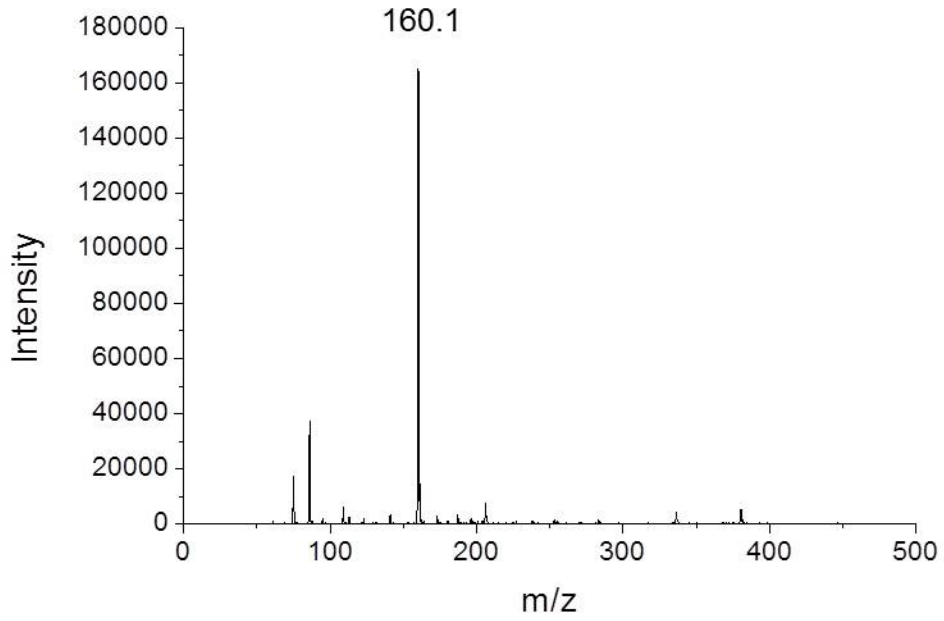

Figure S2. EI Mass-spectrometry spectra of *N*-boc ethanolamine.

^{1}H NMR spectrum of Poly(methyl vinyl ether-*alt*-maleic anhydride)

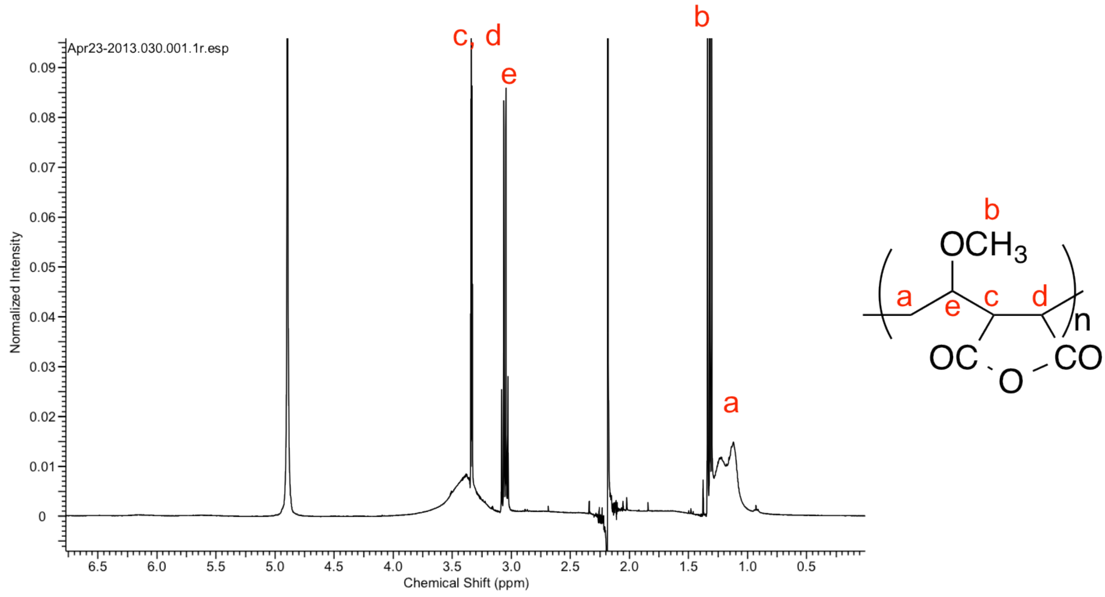

Figure S3. ^{1}H NMR of Poly(methyl vinyl ether-*alt*-maleic anhydride).

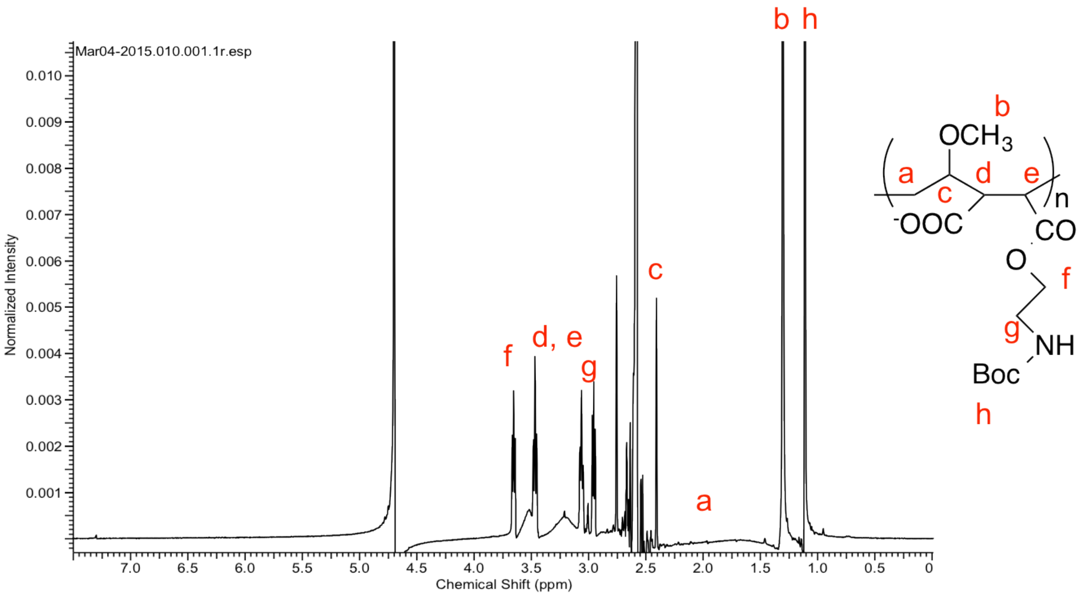

Figure S4. ^{1}H NMR functionalized PMVEMA with boc protected ethanol amine.

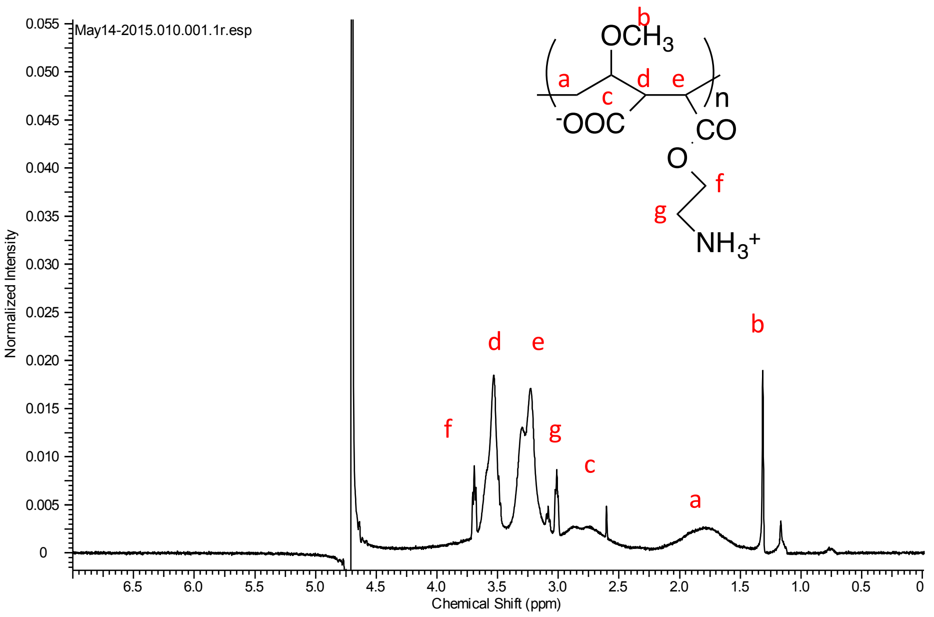

Figure S5. ^{1}H NMR functionalized polymer deprotected with TFA.

Cryopreservation using fast thaw methodology

Samples were frozen in liquid nitrogen, stored for 5 days then thawed at 37 °C in a water bath. The rapid level of thawing limits the time in which ice recrystallization can occur and limits damage to cells, figure S3 shows greater recovery for all samples, and a very limited improvement from adding PMVEMA-*co*-EA. However, the volumes of blood stored would likely be scaled up in commercial storage, meaning that differential warming rates would occur making the slow warming methodology more relevant for cryoprotective applications.

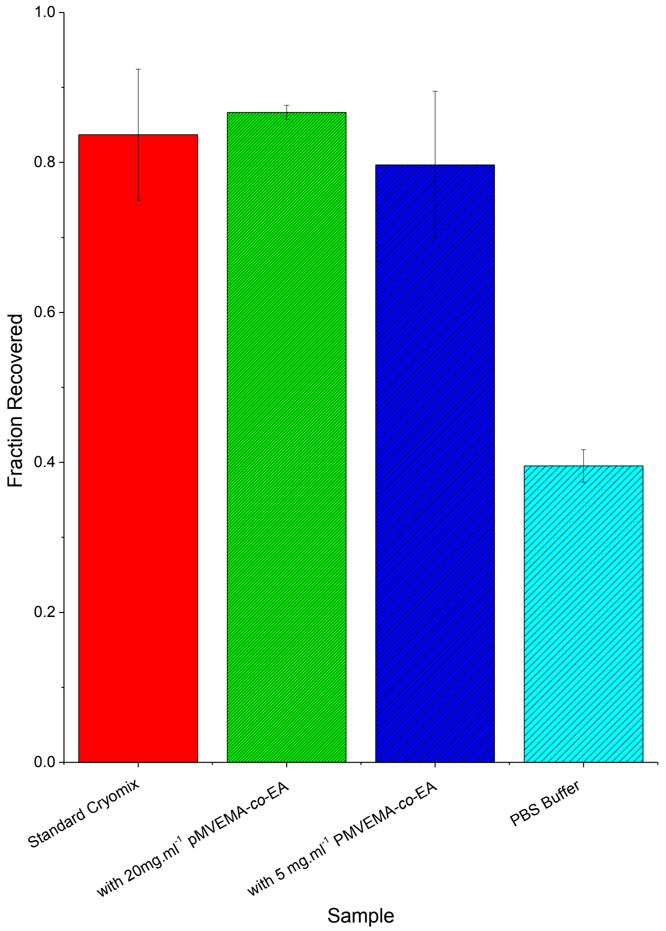

Figure S7. Recovery from fast thawing of red blood cells. Standard Cryomix contained containing HES (350 mg.ml^{-1}), mannitol (30 mg.ml^{-1}) sodium chloride (6.5mg.ml^{-1}). Error bars represent ± SD from a minimum of 3 repeats.

[1] C. A. Knight, J. Hallett, A. L. DeVries, *Cryobiology* **1988**, *25*, 55-60.
